# Plasmon Sensitized Heterojunction 2D Ultrathin Ag/AgI-δ-Bi_2_O_3_ for Enhanced Photocatalytic Nitrogen Fixation

**DOI:** 10.3390/nano9050781

**Published:** 2019-05-22

**Authors:** Xiaoming Gao, Yanyan Shang, Kailong Gao, Feng Fu

**Affiliations:** Department of Chemistry and Chemical Engineering, Shaanxi Key Laboratory of Chemical Reaction Engineering, Yan’an University, Yanan 716000, China; zisusyy@163.com (Y.S.); 17809211625@139.com (K.G.)

**Keywords:** ultrathin, heterojunction, plasmon, photocatalytic nitrogen fixation

## Abstract

A novel 2D ultrathin Ag/AgI-δ-Bi_2_O_3_ photocatalyst was constructed by a facile hydrothermal and in situ photodeposition method, which presented a uniform nanosheet structure with an average height of 6 nm. Its composition, morphology and light-harvesting properties were characterized by X-ray diffraction (XRD), X-ray photoelectron spectroscopy (XPS), scanning electron microscopy (SEM), transmission electron microscopy (TEM), UV–vis spectrophotometer (UV–vis) and photoluminescence (PL) measurements in detail. The Ag/AgI-δ-Bi_2_O_3_ nanocomposites showed an excellent photocatalytic nitrogen fixation performance of 420 μmol L^−1^ g^−1^ h^−1^ in water without any sacrificial agent. The introduction of Ag/AgI nanoparticles caused the morphology modification of δ-Bi_2_O_3_, a higher concentration of oxygen vacancy, and the construction of a plasmon sensitized heterojunction, resulting in enhanced light absorption, improved separation efficiency of charge carriers and strong N_2_ absorption and activation ability, which are responsible for the superior photocatalytic performance of Ag/AgI-δ-Bi_2_O_3_.

## 1. Introduction

Ammonia (NH_3_) is one of the most important chemical products due to its role in natural biological processes as a hydrogen carrier and for the synthesis of many nitrogenous compounds [1]. The traditional artificial ammonia synthesis is accomplished by the Haber–Bosch process using N_2_ and H_2_, which operates at high temperatures and high pressures with high energy input and a high demand for H_2_, producing a serious environmental impact [2]. Therefore, it is highly desirable to realize nitrogen fixation under a mild, cost-effective, and environmentally friendly method.

In recent years, photocatalytic N_2_ fixation driven by solar energy has attracted considerable attention because it uses earth-abundant water as a reductant and can be conducted at atmospheric pressure and room temperature [3,4]. Therefore, a series of materials, namely, g-C_3_N_4_, MoO_3−*x*_, TiO_2_, Ni_2_P/Cd_0.5_Zn_0.5_S, BiOCl, BiOBr, and Bi_5_O_7_Br, have been developed for photocatalytic N_2_ fixation [4,5,6,7,8,9,10,11]. Although numerous studies have been conducted, there is still a bottleneck in developing an ideal photocatalytic nitrogen fixation [5,6,12], such as (1) the indispensable provision of abundant electrons due to six electrons being used for the reduction of one molecule of N_2_, (2) the need to provide higher energy to activate and cleave the triple bond owing to the presence of stable nitrogen molecules, and (3) the necessary requirement of N_2_ adsorption for photocatalytic nitrogen fixation [8,9,11,13,14]. Hence, in order to solve these problems and obtain higher performance of photocatalytic nitrogen fixation, the core issues remain focused on photocatalyst design. As one of the attractive inorganic materials, the *p*-type Bismuth oxide (Bi_2_O_3_) semiconductor has been widely investigated as a promising visible light photocatalytic material with appropriate band gaps [15,16]. In particular, δ-Bi_2_O_3_ was of great interest to our research because it is much richer in anion oxygen vacancies [17,18], which may act as potential candidates for photocatalytic N_2_ fixation. It has been reported that the presence of oxygen vacancies can promote light harvesting and charge carrier separation efficiency, especially improving the adsorption and activation ability of N_2_ in the photocatalytic nitrogen fixation reaction [6,19,20]. However, δ-Bi_2_O_3_ is known to have a high-temperature stable phase of higher than 700 °C [15,17], and it is still a challenge to evolve a facile and effective strategy for the synthesis of δ-Bi_2_O_3_ with high concentrations of oxygen vacancies at a low temperature. Besides, special morphological structure control with a large surface area to provide more active sites should be considered [21]. In addition, the rational construction of a nano-heterojunction is also a feasible route for enhancing photocatalytic performance [22,23]. Recently, the new plasmon resonance photocatalyst silver/silver halides (Ag/AgX, X = Cl, Br, I) attracted much attention when combined with other semiconductor photocatalytic materials, because Ag/AgX hybrid materials dramatically increase visible light utilization and promote photogenerated charge separation [24,25,26]. Simultaneously, Ag/AgX semiconductor materials exhibit aexcellent photocatalytic activity owing to the localized surface plasmon resonance (LSPR) effect, which is described as the resonant photon-induced collective oscillation of valence electrons when the frequency of photons matches the natural frequency of surface electrons oscillating against the restoring force of the positive nuclei [27,28]. So, the combination of δ-Bi_2_O_3_ and Ag/AgX composites could provide new opportunities for developing highly efficient visible-light driven nitrogen fixation photocatalysts due to the LSPR effect and the heterostructure. Up until now, there have been few reports on photocatalytic N_2_ fixation application by δ-Bi_2_O_3_ based photocatalysts with well-designed morphology and heterojunction structure.

In this work, we developed a facile hydrothermal method to synthesize δ-Bi_2_O_3_ microcrystals at a low temperature, and a Ag/AgI-δ-Bi_2_O_3_ photocatalyst was further prepared via a precipitation and photoreduction route, which was then used as the photocatalyst for nitrogen fixation which achieved remarkably improved N_2_ photoconversion activity compared to that of a pure δ-Bi_2_O_3_ photocatalyst. Meanwhile, the influences of the ultrathin nanosheets structure of the δ-Bi_2_O_3_ and Ag/AgI combination on photocatalytic activity were systematically discussed. The mechanism of photocatalytic nitrogen fixation of the Ag/AgI-δ-Bi_2_O_3_ photocatalyst was also proposed according to the results of systematic analysis, such as photoluminescence (PL), alternating current (AC) impedance, photocurrent response, and fluorescence life and DMPO (5,5-dimethyl-1-pyrroline-N-oxide)–electron spin resonance (ESR).

## 2. Experimental Section

### 2.1. Synthesis of Photocatalysts

A total of 0.4851 g Bi(NO_3_)_3_·5H_2_O was dissolved in a mixed solution containing 8 mL ethylene glycol and 32 mL tert-butyl alcohol, followed by vigorous stirring. Subsequently, the mixture solution was put into a Teflon-lined stainless steel autoclave with a capacity of 50 mL. The autoclave was heated and maintained at 160 °C for 8 h, cooled to room temperature, and dried in a vacuum at 80 °C for 48 h. The mixture solution was formed by adding 0.466 g of δ-Bi_2_O_3_, 0.2 mL (0.5 mL, 1 mL, 3 mL, 5 mL) AgNO_3_ solution (0.01 mol/L), and 0.1 mL (0.25 mL, 0.5 mL, 1.5 mL, 2.5 mL) KI solution (0.01 mol/L) into deionized water. Afterwards, the above solution was stirred for 1 h and then illuminated by 400 W gold halide lamp for 2 h. Then, the samples were washed by deionized water and dried in a vacuum at 80 °C for 48 h. The as-prepared samples were named Ag/AgI–BiO-1, Ag/AgI–BiO-2, Ag/AgI–BiO-3, Ag/AgI–BiO-4, and Ag/AgI–BiO-5 (0.2%Ag/AgI-δ-Bi_2_O_3_, 0.5%Ag/AgI-δ-Bi_2_O_3_, 1%Ag/AgI-δ-Bi_2_O_3_, 3%Ag/AgI-δ-Bi_2_O_3,_ and 5%Ag/AgI-δ-Bi_2_O_3_), respectively.

### 2.2. Characterization

The composition and crystal phase of the samples was identified by 7000 powder X-ray diffraction (Shimadzu, Tokyo, Japan) using an X-ray diffractometer with Cu-Kα radiation under 40 kV and 30 mA. The X-ray photoelectron spectroscopy of the as-prepared samples was performed on an ESCALAB 250 Xi X-ray photoelectron spectrometer (ThermoScientific, Waltham, MA, USA). The morphologies of the samples were shown on a JSM-6700F scanning electron microscope (JEOL, Tokyo, Japan). The determination of element composition was measured using an energy dispersive spectrometer (Bruker, Bremen, Germany). High-resolution transmission electron images of the as-prepared samples were measured on a JEM-2100 electron microscope (JEOL, Tokyo, Japan). The ultrathin structure of the as-prepared sample was determined by Cypher S atomic force microscopy (Oxford, UK). The UV–vis diffuse reflectance spectroscopy (DRS) was measured by a UV–2550 spectrophotometer (Shimadzu, Tokyo, Japan) with BaSO_4_ as a reference. The photo–electro–chemical properties of the samples were collected on a CHI660D electrochemical workstation (Chenhua, Shanghai, China). An F-4500 fluorescence spectrograph (Hitachi, Tokyo, Japan) was used to analyze the fluorescence spectra of samples. The Brunauer–Emmett–Teller specific surface area was analyzed by a V-Sorb2800P analyzer using adsorption and desorption of N_2_, and N_2_ temperature-programmed desorption (TPD) was performed by 3 Flex Chemi instrument (Micromeritics, Norcross, GA, USA). The electron paramagnetic resonance (EPR) spectra of the samples were determined with an ES-ED3X Endor spectrometer (JEOL, Tokyo, Japan).

### 2.3. Photocatalytic Reaction

Photocatalytic nitrogen fixation experiments were carried out on a quartz glass photochemical reactor under the irradiation of a 400 W xenon lamp with a 420 nm cutoff filter. A total of 0.2 g photocatalyst was dispersed in 200 mL of deionized water, and nitrogen flow was kept bubbling through the solution at a rate of 10 mL/min. Before irradiation, the mixture solution was stirred constantly in the dark for 30 min. Then, 5 mL of aqueous solution was taken out after 30 min, and the NH_4_^+^ concentration was determined by a Metrohm 833 Ions Chromatography (Herisau, Switzerland).

## 3. Results and Discussion

**Figure 1:**
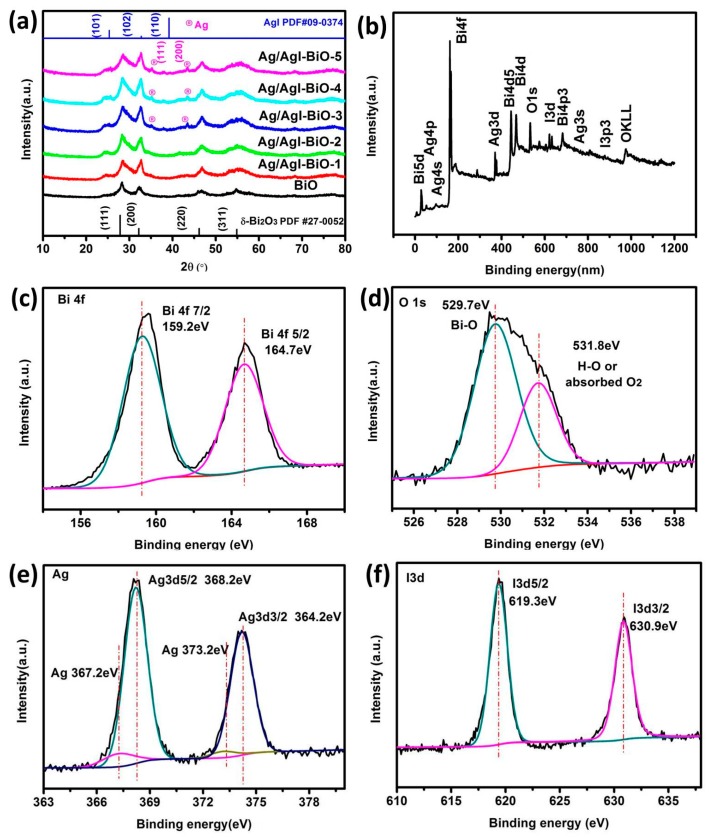
(**a**) X-ray diffraction (XRD) of as-prepared samples; (**b**) The survey spectra and the high-resolution X-ray photoelectron spectroscopy (XPS) spectra of Ag/AgI–BiO-4; (**c**) Bi 4f; (**d**) O1s; (**e**) Ag3d; (**f**) I3d.

**Figure 2:**
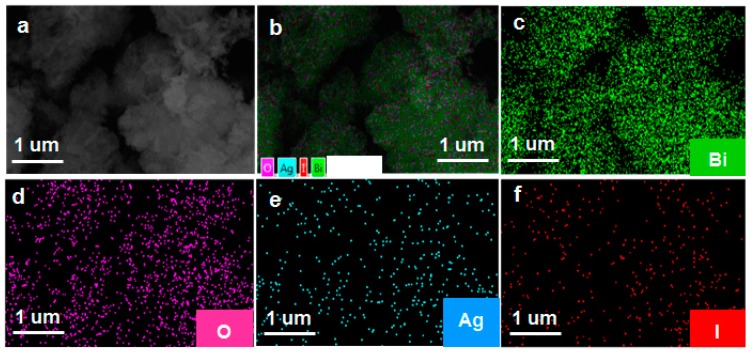
(**a**) The scanning electron microscopy (SEM); (**b**) the total element distribution; and (**c**–**f**) the element distribution of Bi, O, Ag, and I of 3%Ag/AgI-δ-Bi_2_O_3_.

The phase structure of the as-prepared samples was characterized by the XRD. As shown in Figure 1a, the diffraction peaks located at 28.0°, 32.3°, 46.3°, and 55°, corresponded to the crystal planes of (111), (200), (220), and (311), and can be indexed to the cubic planes of δ-Bi_2_O_3_ (JCPDS No. 27-0052, Pn3m (224), *a* = *b* = *c* = 5.525 Å). The diffraction peaks located at 25.35°, 32.76°, and 39.20°, corresponded to the crystal planes of (101), (102) and (110), and can be assigned to β-AgI (JCPDS No.09–0374). As far as Ag/AgI–BiO-3, Ag/AgI–BiO-4 and Ag/AgI–BiO-5 are concerned, there were two main diffraction peaks at 38.12° and 44.28°, corresponding to the crystal planes of (111) and (220) of Ag (JCPDS No.04–0783). It is implied that Ag deposited on the surface of BiO can be detected from the added content of Ag. From the unit cell parameter of the as-prepared samples (Table 1), the cell volume of Ag/AgI–BiO changed slightly in comparison with BiO, indicating that the Ag/AgI deposited was only deposited on the surface of BiO, and did not change the original lattice of δ-Bi_2_O_3_. The surface chemical composition of the as-prepared sample was measured by X-ray photoelectron spectroscopy (XPS). As shown in the survey spectra of Ag/AgI–BiO-4 (Figure 1b), the sample was composed of Bi, O, Ag, and I, which confirmed the chemical composition of the as-prepared sample. From the narrow spectrum of the Bi element (Figure 1c), there were two peaks at 159.2 and 164.7 eV, ascribed to the characteristic peaks of Bi4f_7/2_ and Bi 4f_5/2_, respectively, which indicated that Bi existed in the as-prepared sample in the form of Bi^3+^. Two obvious characteristic peaks at 529.7 and 532.8 eV were observed in the narrow spectrum of the O element (Figure 1d), which corresponded to the Bi–O band and the absorbed O_2_ or H–O bond, respectively. From Figure 1e, the two characteristic peaks that appeared in the 368.2 and 374.2 eV corresponded to the Ag 3d_5/2_ and Ag 3d_3/2_, indicating the existence of Ag^+^; meanwhile, the characteristic peaks that appeared at 367.2 and 373.2 eV showed the presence of Ag^0^ on the surface of the sample. The peaks at 619.3 and 630.9 eV can be assigned to the characteristic peaks of I3d_5/2_ and I3d_3/2_ (Figure 1f). An energy dispersive spectrometer (EDS) result indicated that the elements of Bi, O, Ag, and I were well distributed on the surface of the photocatalyst (Figure 2).

**Figure 3:**
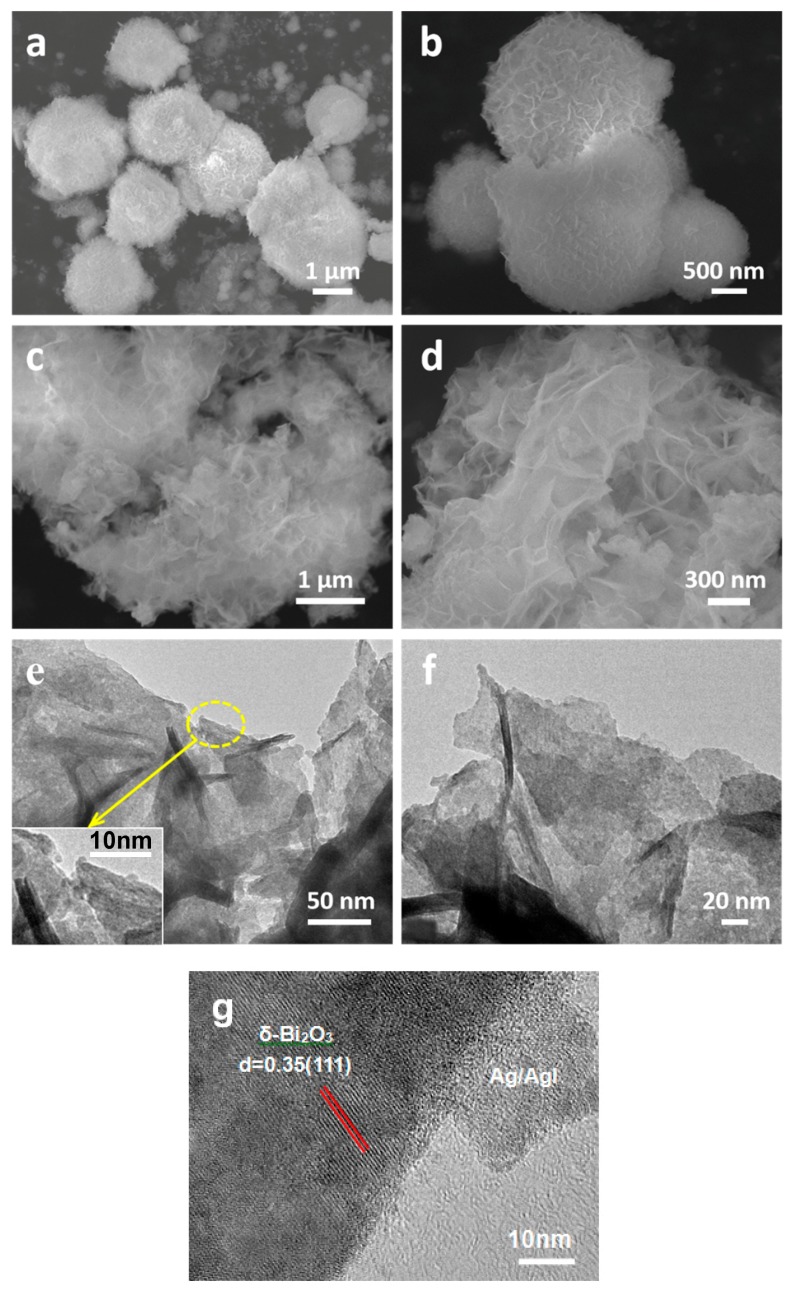
(**a**,**b**) The SEM images of pure δ-Bi_2_O_3_ sample, and (**c**,**d**) SEM, (**e**,**f**) transmission electron microscopy (TEM) images and (**g**) the high resolution TEM images of Ag/AgI–BiO-4.

The morphology and microstructure of pure δ-Bi_2_O_3_ and Ag/AgI–BiO-4 were observed by scanning electron microscopy (SEM) and transmission electron microscopy (TEM). As presented in Figure 3a,b, the pure δ-Bi_2_O_3 (_without modification) displayed a microsphere hierarchically structure, which was built from numerous pliable nanosheets aligned to the spherical surface. However, it was observed that the structure of δ-Bi_2_O_3_ turned into an ultrathin two-dimension layered nanosheet morphology after the deposition of Ag/AgI nanoparticles by mild precipitation and photoreduction reaction processes (as shown in Figure 3c,d). This remarkable change may be achieved by the photo-assisted Ag exfoliation effect [29]. In such a specific structure, the multilayer nanosheets do not only provide more exposure space for photocatalytic reaction but can also promote the photogenerated electron transport within the photocatalyst. The photogenerated carriers are more easily transported to the surface of the photocatalyst to participate in chemical reactions after irradiation, thus further reducing the recombination rate of photogenerated electrons and holes. However, the Ag/AgI nanoparticles failed to be observed in SEM images due to their small size and content. More detailed structural information of the Ag/AgI–BiO-4 composite was further revealed by TEM images, as presented in Figure 3e,f, where Ag/AgI–BiO-4 presented as a 2D ultrathin nanosheet structure with a size of hundreds nanometers, and many small nanoparticles of about several nanometers corresponding to Ag and AgI were deposited on the surface of the δ-Bi_2_O_3_ nanosheet. From the high resolution transmission electron microscopy (HR–TEM) of Ag/AgI–BiO-4 (Figure 3g), clear lattices with spaces of 0.35 nm, corresponding to the (111) facets of δ-Bi_2_O_3_ and the deposited phase of Ag/AgI, were observed clearly. 

**Figure 4:**
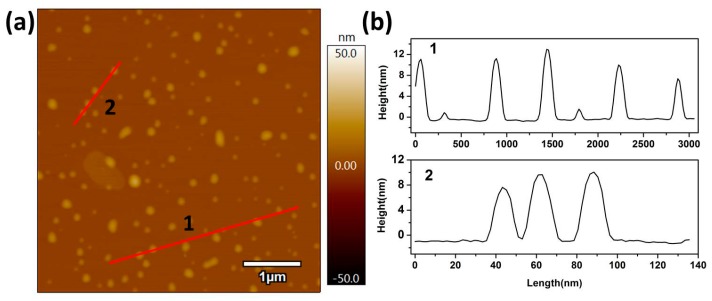
(**a**) Atom force microscopy (AFM) image and (**b**) the corresponding height of Ag/AgI–BiO-4.

Furthermore, the height of the single nanosheet was observed by atom force microscopy (AFM), as shown in Figure 4, where the average height of the single nanosheet was approximately 6 nm. However, a thickness of about 12 nm was also found, which implied the stacking of two single nanosheets. Generally, the especially ultrathin nanosheets and nanoparticle (<10 nm) structures of the Ag/AgI–BiO composite display unusual physical and chemical properties due to prominent quantum surface effects and distinct electronic structures.

**Figure 5:**
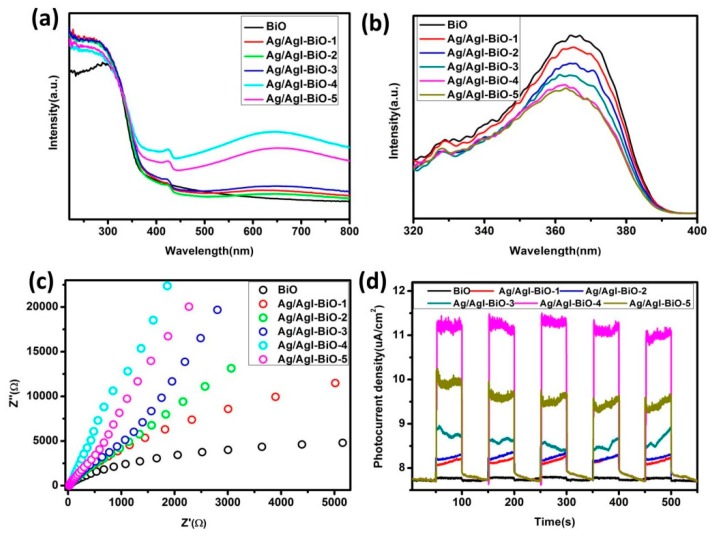
(**a**) The UV–vis diffuse reflectance spectroscopy (DRS) of as-prepared samples; (**b**) the photoluminescence (PL) of the as-prepared samples; (**c**) the Nyquist spectra of alternating current (AC) impedance of the as-prepared samples; (**d**) the photocurrent of the as-prepared samples.

The light absorption and optical response range of the as-prepared samples was investigated by the UV–vis DRS. As shown in Figure 5a, all of the as-prepared samples had evident absorption from the UV to visible range. In comparison with BiO, the red shift was not obvious for Ag/AgI deposited BiO, but the as-prepared Ag/AgI–BiO samples appeared to have obvious enhancement of visible light absorption, which is mainly attributed to the strong surface plasmon resonance of Ag nanocrystals and the synergistic effect between Ag/AgI and δ-Bi_2_O_3_ [24,30], resulting in better visible light utilization. The recombination efficiency of the photogenerated carriers of the as-prepared samples was investigated by photoluminescence spectroscopy. From Figure 5b, the fluorescence emission intensity of Ag/AgI–BiO was lower than that of pure BiO, indicating that the deposition of Ag/AgI can obviously reduce the recombination rate of the photogenerated carriers. In comparison with the Ag/AgI–BiO, Ag/AgI–BiO-4 and Ag/AgI–BiO-5 have nearly the same lowest fluorescence emission intensity, indicating that the energy loss of the recombination of the photogenerated carriers is weak. The transmission speed of the photogenerated carrier of the catalysts was measured by the electrochemical impedance spectroscopy, which reflected the charge transfer resistance between the solid interface and surface, and the smaller the arc radius of the Nyquist spectra of electrochemical impedance, the easier the separation of the photogenerated carrier. As shown in Figure 5c, the electrochemical impedance spectrum of Ag/AgI–BiO-4 had the lowest arc radius, which implies a faster transmission speed and a more efficient separation for the interfacial transfer of the photogenerated carrier. The photocurrent density can be used to explain the separation efficiency of the photogenerated carrier. As shown in Figure 5d, the transient photocurrent of the as-prepared samples presented stable cycles with a light on or off. The BiO had a weaker light current response of 0.11 μA cm^−1^, and the Ag/AgI–BiO-4 owned the largest photocurrent density, which was about 3.74 μA cm^−1^. The significantly enhanced photocurrent density implies the significant improvement of separation efficiency of the photogenerated carriers. The fluorescence lifetime of the photogenerated carriers of the as-prepared samples was measured by time-resolved fluorescence spectroscopy. As shown in Table 2, the fitting factor χ^2^ was close to 1. The fluorescence life of BiO was 0.192 ns, which was shorter than that of Ag/AgI–BiO. Especially, Ag/AgI–BiO-4 possessed the longest fluorescence life of 0.765 ns. The prolonged fluorescence lifetime indicates a slow recombination rate of the photogenerated carriers.

**Figure 6:**
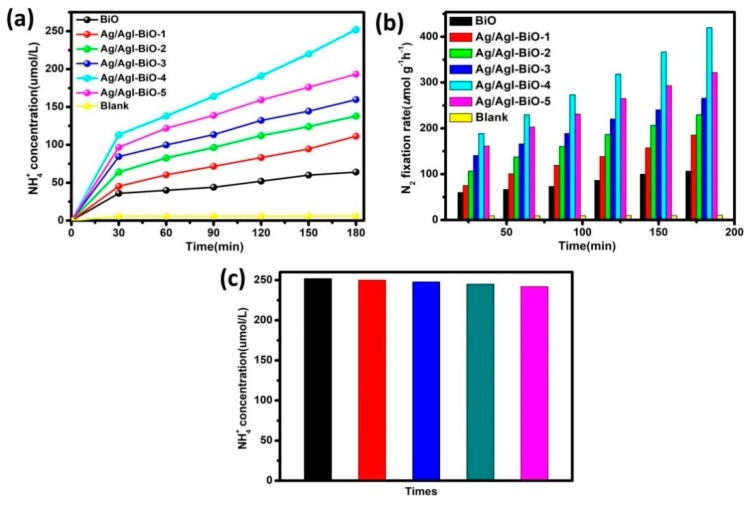
(**a**) NH_4_^+^ concentration in water; (**b**) photocatalytic nitrogen fixation rate over the different catalysts; and (**c**) effect of recycling use of Ag/AgI–BiO-4 to the NH_4_^+^concentration in water.

The photocatalytic nitrogen fixation efficiencies were tested using water as the proton source without any scavenger under light irradiation. As shown in Figure 6, under normal temperature and atmospheric pressure conditions, the NH_4_^+^ in water was almost undetectable under light irradiation and bubbling nitrogen. However, all samples exhibited a near linear increase of nitrogen fixation performance over the entire reaction time under light irradiation, suggesting good stability during the photocatalytic N_2_ fixation process. After visible light irradiation for 3 h in the case of pure BiO, the NH_4_^+^ concentration in water was about 64 μmol/L (Figure 6a), and the NH_4_ fixation rate was 107 μmol L^−1^ g^−1^ h^−1^ (Figure 6b). In comparison, a significant improvement in the photocatalytic nitrogen fixation activities was observed with the addition of Ag/AgI, and the Ag/AgI–BiO-4 nanocomposite exhibited the highest nitrogen fixation rate of 420 μmol L^−1^ g^−1^ h^−1^, with the corresponding amount of NH_4_^+^ reaching 250 μmol/L, which was four times higher than that of BiO. The apparent quantum efficiency (AQE) was measured under the same reaction conditions except in incident light, which is 420 nm monochromatic light. The NH_3_ amount was measured after 1 h of irradiation, and the AQE calculated as the reference [4,9]. The AQE of Ag/AgI–BiO-4 (4.1%) was approximately two times than that of BiO (2.0%). This enhancement in the nitrogen fixation rate can be attributed to the two-dimensional ultrathin nanosheet morphology of Ag/AgI–BiO with its higher specific surface area and more active sites for promoting surface nitrogen fixation kinetics. Meanwhile, as characterized in UV–vis and photoelectrochemical tests, the enhanced light harvesting capacity and the improved carrier separation efficiency caused by the introduction of Ag/AgI are important reasons for enhanced photocatalytic performance. In addition, the recycle experiments for photocatalytic nitrogen fixation were carried out, and the results are shown in Figure 6c. After being used five times, the NH_4_^+^ concentration over Ag/AgI–BiO-4 was virtually identical, which indicates that the catalyst can be continuously applied in photocatalytic N_2_ fixation.

**Figure 7:**
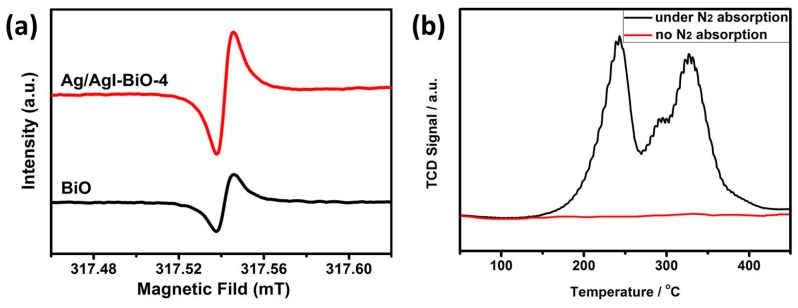
(**a**) Low-temperature EPR spectra and (**b**) the N_2_-TPD of Ag/AgI–BiO-4.

Furthermore, EPR was performed to analyze the concentration of oxygen vacancies in the photocatalysts. As shown in Figure 7a, two obviously symmetrical EPR signals at the g value of 2.00 were observed, which were identified as the electrons trapped on the oxygen vacancies [31,32]. It was observed that the EPR intensity of Ag/AgI–BiO-4 was significantly stronger than that of BiO, which demonstrated a higher surface oxygen vacancy concentration for Ag/AgI–BiO-4. This phenomenon probably occurs because of the morphological modification of the δ-Bi_2_O_3_ ultrathin nanosheet with more exposed surfaces owing to the photo-assisted Ag exfoliation effect. Besides, it is known that the oxygen vacancies can promote the adsorption and activation of N_2_ in a more feasible pathway, so N_2_-TPD was carried out to understand the N_2_ chemical adsorption on the surface of photocatalysts. As shown in Figure 7b, the two desorption peaks at 242 and 328 °C corresponded to the physical adsorption and chemical adsorption of N_2_, respectively, and the Ag/AgI–BiO-4 photocatalyst displayed a much higher adsorption ability compared to BiO, which is consistent with photocatalytic N_2_ fixation activity. These results suggest that the combination of Ag/AgI can introduce more oxygen vacancies in a catalyst system, which benefits the conversion efficiency of nitrogen photofixation.

**Figure 8:**
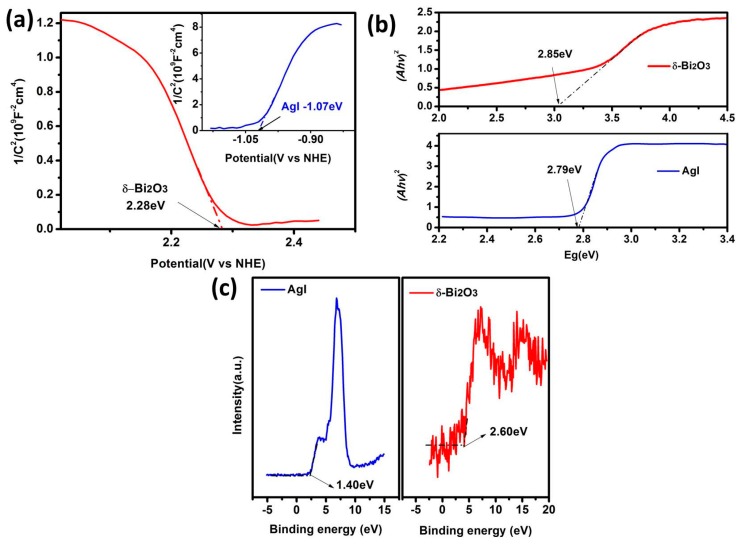
(**a**) Mott–Schottky plots (**b**) band gap energy and (**c**) valence-band (VB) XPS spectra of AgI and δ-Bi_2_O_3._

In order to explain the photocatalytic mechanism, the energy band structures of δ-Bi_2_O_3_ and AgI were further determined by Mott–Schottky analysis and valence-band XPS spectra. The Mott–Schottky plots of δ-Bi_2_O_3_ and AgI are shown in Figure 8a. According to the intercept of the plots on the abscissa axis, the *E*_fb_ vs. NHE (normal hydrogen electrode) of δ-Bi_2_O_3_ and AgI were calculated to be 2.28 and −1.07 eV, respectively. The negative slope of the δ-Bi_2_O_3_ confirmed its *p*-type semiconductivity, and the AgI showed an *n*-type semiconductor character. Besides, as it is known that the *E*_fb_ is about 0.3 eV above valence-band (VB) potential for *n*-type semiconductor and 0.3 eV below the conduction-band (CB) potential for *n*-type semiconductors [33,34], we can conclude that the *E*_VB_ of δ-Bi_2_O_3_ is calculated as 2.58 eV vs. NHE and that the *E_C_*_B_ of AgI is determined as −1.37 eV vs. NHE. Additionally, as shown in Figure 8b, the band gap energy can be estimated from the intercept of the UV–vis DRS plots, which were 2.85 eV for δ-Bi_2_O_3_ and 2.79 eV for AgI, respectively. Consequently, *E*_CB_ of δ-Bi_2_O_3_ can be calculated as −0.27 eV, and *E*_VB_ of AgI at approximately 1.42 eV—those values agreeing with the VB XPS spectra (Figure 8c). Due to the direct contact between δ-Bi_2_O_3_ and AgI, a heterojunction with band matching can be established due to the staggered band alignment, where an electric field can form at the interface to promote the electron-hole separation and migration, which is in favor of photocatalytic N_2_ fixation.

**Figure 9:**
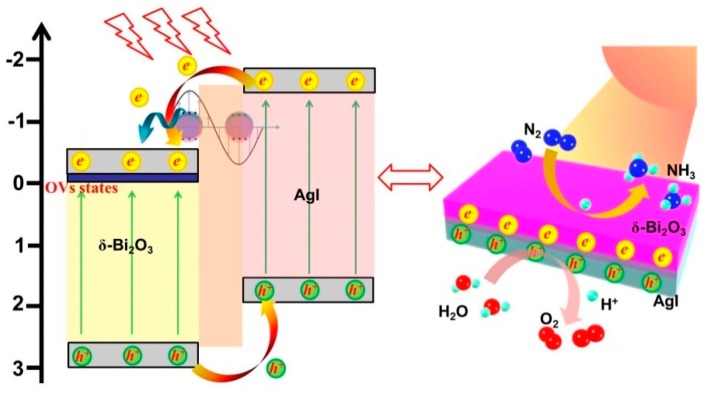
The possible photocatalytic degradation mechanism.

Based on these results, the reasons for the enhanced photocatalytic activity and probable photogenerated charge carriers transport mechanism of the Ag/AgI–BiO nanocomposite were supposed. Due to the introduction of Ag/AgI, δ-Bi_2_O_3_ exhibited a great change in morphology as ultrathin nanosheets, which provided more reactive sites for N_2_ fixation. More importantly, the concentration of surface oxygen vacancies showed a significant increase to facilitate the adsorption and activation of inert N_2_ molecules. As shown in Figure 9, under light irradiation, electrons and holes were generated on the CB and VB of AgI and δ-Bi_2_O_3_. On account of the heterojunction characteristic and the staggered band alignment structure between δ-Bi_2_O_3_ and AgI, photoinduced holes tended to migrate from δ-Bi_2_O_3_ to AgI, and the photoexcited electrons of AgI were transferred to δ-Bi_2_O_3_ and then trapped by oxygen vacancies with a defective state. Simultaneously, the metal Ag nanoparticles absorbed photons to form free electrons with higher energy states owing to the LSPR effect, and the hot electrons migrated from Ag to δ-Bi_2_O_3_ subsequently [35]. Therefore, photogenerated electrons and holes were effectively separated. The photogenerated electrons were subsequently consumed by adsorbing N_2_ molecules and H^+^ to form NH_4_^+^, and the photogenerated holes participated in the oxidation of H_2_O to O_2_. According to the above analyses, more activated nitrogen and electrons can be supplied for the photocatalytic reaction over the Ag/AgI–BiO system, resulting in a superior photocatalytic nitrogen fixation performance.

## 4. Conclusions

In summary, we have developed a facile hydrothermal–photodeposition strategy to prepare 2D ultrathin Ag/AgI-δ-Bi_2_O_3_ photocatalysts with a highly efficient photocatalytic N_2_ fixation activity, which exhibits obviously superior photocatalytic activity with a N_2_ fixation rate of about 420 μmol L^−1^ g^−1^ h^−1^ compared with δ-Bi_2_O_3_ photocatalysts. On the basis of detailed characterizations and tests, the morphology modification of δ-Bi_2_O_3_ resulted in a bigger specific surface area and more exposed oxygen vacancies, with the enhanced photocatalytic performance of Ag/AgI-δ-Bi_2_O_3_ nanocomposite being attributed to the increased visible light absorption, the improved carrier separation efficiency forming the plasmonic effect, and the heterojunction and enhanced N_2_ adsorption and activation caused by oxygen vacancies. This work offers a novel approach to the design and construction of high-efficiency Bi_2_O_3_ based photocatalysts in the field of solar-driven ammonia synthesis.

## Figures and Tables

**Table 1:** The unit cell parameter of the as-prepared catalyst.

Sample	Unit Cell Volume (Å^3^)	*a* (Å)	*b* (Å)	*c* (Å)
δ-Bi_2_O_3_	168.65	5.525	5.525	5.525
0.2%Ag/AgI-δ-Bi_2_O_3_	168.56	5.524	5.524	5.524
0.5%Ag/AgI-δ-Bi_2_O_3_	168.38	5.522	5.522	5.522
1%Ag/AgI-δ-Bi_2_O_3_	168.20	5.520	5.520	5.520
3%Ag/AgI-δ-Bi_2_O_3_	168.83	5.527	5.527	5.527
5%Ag/AgI-δ-Bi_2_O_3_	168.92	5.528	5.528	5.528

**Table 2:** The fluorescence life of the as-prepared samples.

Sample	τ_1_	τ_2_	χ^2^	τ
BiO	0.1/94.93	1.92/5.07	1.194	0.192
Ag/AgI–BiO-1	0.37/96.74	1.39/3.24	1.129	0.403
Ag/AgI–BiO-2	0.38/94.58	1.47/5.42	1.135	0.439
Ag/AgI–BiO-3	0.42/90.25	1.53/9.47	1.112	0.524
Ag/AgI–BiO-4	0.57/84.53	1.83/15.47	1.092	0.765
Ag/AgI–BiO-5	0.49/88.53	1.56/11.47	1.104	0.613
